# Reconstruction of Total Bone Defects following Resection of Malignant Tumors of the Upper Extremity with 3D Printed Prostheses: Presentation of Two Patients with a Follow-Up of Three Years

**DOI:** 10.1155/2020/8822466

**Published:** 2020-10-02

**Authors:** Thomas Ackmann, Sebastian Klingebiel, Georg Gosheger, Anna Rachbauer, Christoph Theil, Dimosthenis Andreou

**Affiliations:** ^1^Department of General Orthopedics and Tumor Orthopedics, Muenster University Hospital, Muenster, Germany; ^2^Division of Orthopedic Oncology and Sarcoma Surgery, Helios Klinikum Bad Saarow, Bad Saarow, Germany

## Abstract

Wide tumor resection is the local treatment of choice for patients with primary malignant bone tumors and a prerequisite for long-term survival. We present two patients that underwent total bone resection in the upper limb because of primary malignant bone tumors. The defects were then reconstructed by a 3D printed prosthesis, a procedure that, to our knowledge, has not been reported for bone defects of the upper extremity so far. Complete resection of the affected bone was required in a five-year-old girl with a high-grade osteoblastic osteosarcoma of the humerus and a 53-year-old man with a dedifferentiated leiomyosarcoma of the radius, due to the tumor's extent. Following neoadjuvant chemotherapy, resection of the entire affected humerus including the axillary nerve took place in the first case and the entire affected radius including parts of the radial nerve in the second case. Approximately three years after surgery, both patients are alive and pain-free. Despite a postoperative drop hand that affected the now 56-year-old man, he is able to carry out everyday activities such as brushing his teeth, writing, and eating. The now eight-year-old girl is also able to engage in normal activities with her left arm such as eating and carrying lightweight objects. Both patients are tumor-free to date.

## 1. Introduction

Wide tumor resection is the local treatment of choice for patients with primary malignant high-grade bone tumors and a prerequisite for long-term survival [1]. This involves the en bloc removal of the tumor with a safety margin of healthy bone and soft tissue. In some cases, a large longitudinal tumor extent or the presence of skip metastases can render the resection of the entire affected bone unavoidable [2].

In the long bones of the extremities, the resulting defects are most commonly reconstructed with modular endoprothesis or biological reconstruction methods [2–4]. The choice of the optimal method depends on a variety of factors, such as the location and length of the defect, joint involvement, patient age, expected prognosis, and whether adjuvant chemo- and radiotherapy are necessary [5].

An innovative method is the reconstruction with custom-made 3D printed prostheses. This method has already been described for patients with clavicle Ewing's sarcoma, scapular Ewing's sarcoma, pelvic chondrosarcoma, and calcaneal chondrosarcoma [6, 7] However, to our knowledge, no reports on the clinical application of customized 3D printed prostheses in the long bones of the upper extremity are available in the literature. We therefore present two cases of total bone resection in the upper limb and reconstruction with 3D printed prostheses constructed by additive manufacturing.

## 2. Case Presentation

**Figure 1:**
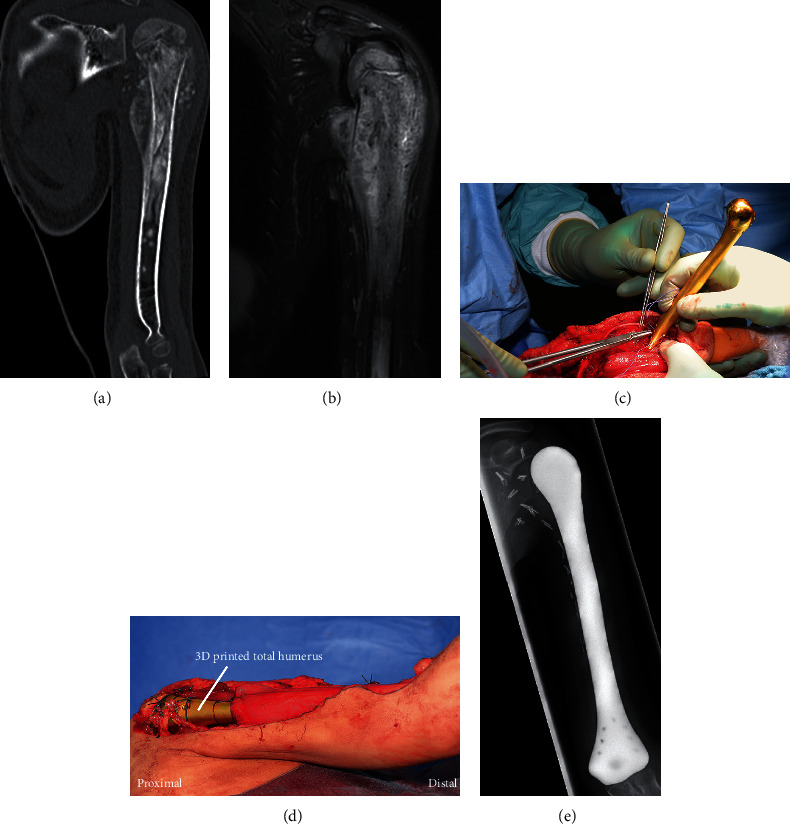
A five-year-old girl with a high-grade osteosarcoma of the left proximal humerus. (a, b) CT and MRI scans showed a locally advanced tumor involving almost the complete humerus. (c, d) Reconstruction of the osseous defect by the 3D printed total humerus. (e) X-ray image one year after surgery showed no dislocation.

A five-year-old girl with a high-grade osteoblastic osteosarcoma of the humerus (Figures 1(a) and 1(b)) and a 53-year-old man with a dedifferentiated leiomyosarcoma of the radius underwent complete excision of the affected bone, necessary due to the tumors' extent.

**Figure 2:**
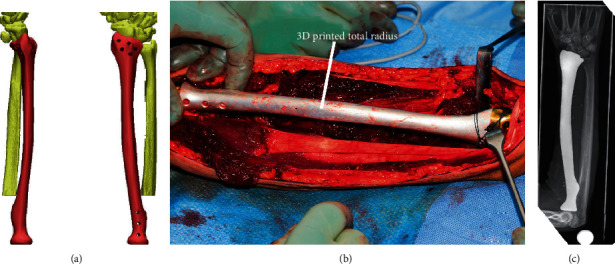
A 53-year-old man with a dedifferentiated leiomyosarcoma of the radius. (a) Virtual three-dimensional model of the radius. (b) Reconstruction with the 3D printed total radius. (c) X-ray image almost one year after implantation.

In order to construct the 3D printed prosthesis, a low-dose CT scan (Siemens®, Somatom Definition AS) of the corresponding contralateral bone was initially performed. The data was stored in Digital Imaging and Communication in Medicine (DICOM®) format. The virtual 3D bone image (Figure 2(a)) was digitally mirrored and cut into thin slices. The thickness of each slice amounted to about 50 *μ*m, which corresponded to the thickness of each layer of the additive manufacturing method, which was applied. The powder application (titanium alloy with 6% aluminum and 4% vanadium) then followed and a uniform powder layer, equal to a virtual slice was applied and got preheated at 400°C. An electron beam selectively illuminated the area of the powder, where the implant should be formed, on calculated paths and made it melt by converting kinetic energy to thermal energy. The melt solidified and thus established a firm connection with the underlying layer. Next, the worktop was lowered by a layer thickness and the workflow recommenced—a method called additive manufacturing. The production time depended largely on the construction height and lasted about 45 seconds per layer.

Both patients had no evidence of distant metastases in staging. The five-year-old girl underwent neoadjuvant polychemotherapy analog to the EURAMOS-1 protocol from July to September 2016. The 53-year-old man underwent three cycles of neoadjuvant chemotherapy according to the EURO-B.O.S.S. protocol from July to November 2016. Local treatment followed in both cases, involving the resection of the entire affected humerus including the axillary nerve in the first case and the entire affected radius including parts of the radial nerve in the second case. The resulting defects were then reconstructed with 3D printed prostheses (Figures 1(c), 1(d), and 2(b)), which were constructed as described above. The proximal and distal anchorage of the hemiprotheses was performed with tendons, and remaining healthy periarticular soft tissues which were attached to the implants through holes drilled specifically for this reason during manufacturing.

Inpatient treatment after surgery amounted to two weeks for the girl. Her left arm was immobilized after surgery with a shoulder bandage (Donjoy-Ultrasling®) for four weeks. Histological evaluation of the surgical specimen showed a grade IV response to neoadjuvant treatment according to the classification established by Salzer-Kuntschick et al. [8] and adjuvant polychemotherapy analog to the EURAMOS-1 protocol was performed.

The right forearm of the man was fixed in a plaster splint for six weeks after surgery, after which time he received an orthesis due to a drop hand and physical as well as occupational therapy commenced. He spent 13 days in hospital after surgery. Histological examination demonstrated a poor response to neoadjuvant chemotherapy, so he underwent adjuvant treatment according to the high-risk arm of the EURO-B.O.S.S. protocol.

On both cases, no wound healing disorders were observed and X-ray image showed proper positioning (Figures 1(e) and 2(c)) of the prostheses after surgery and during the regular follow-up examinations.

About three years after surgery, both patients are alive and pain free. Despite a postoperative drop hand that affected the now 56-year-old man, he is able to carry out everyday activities such as brushing his teeth, writing, and eating. The now eight-year-old girl is also able to engage in normal activities with her left arm such as eating and carrying lightweight objects. Both patients are tumor-free up to date.

## 3. Discussion and Conclusion

Almost 90% of bone sarcomas arising in the extremities can be treated effectively with limb-salvage surgery, even in case of a large tumor volume [7, 9]. However, there is still a need for innovative reconstruction options, especially in young patients and locally advanced tumors of the upper limb. Lu et al. have demonstrated good clinical results for the reconstruction of intercalary massive bone defects of the femur and tibia in sarcoma patients with 3D printed prostheses combined with beta-triacalcium phosphate bioceramics and/or vascularized fibulae [10], while Fan et al. have reported good results of customized 3D printed titanium prostheses of the clavicle, scapula, and pelvis [2]. Our cases are the first that describe the results of 3D printed prostheses in the long bones of the upper extremity after resection of the entire affected bone. The prostheses were successfully fixated in situ by reattachment of muscles, tendons, and other healthy soft tissues. Similar to the results of Lu et al. and Fan et al., our implants fitted the anatomy of the individual patients well and provided a satisfactory outcome.

High production costs and the long production time are notable disadvantages of this procedure. However, the production costs should be offset by the preservation of a functional limb. Furthermore, the production time poses no problem in patients who need neoadjuvant chemotherapy—as was the case in our series—but may be a limiting factor in patients needing primary surgery or patients with an early joint infection who may be candidates for one stage-exchange revision arthroplasty. Furthermore, the relatively short follow-up of our report precludes any definitive conclusions regarding implant stability and survival.

On the other hand, the procedure offers several advantages beyond being a novel reconstruction method for limb-sparing surgery. The virtual 3D model, which is generated during prosthesis construction, allows the surgeon to evaluate all aspects of the implant preoperatively and assess possible fixation sites. This planning may reduce operation time as it renders the transition of the planning to the patients' surgical procedure more precise [11]. Furthermore, patients are able to see and interact with the virtual 3D model preoperatively, which improves their understanding and possibly acceptance of the procedure.

In conclusion, both cases described in our report demonstrate that reconstruction of total bone defects in the upper limb with 3D printed prostheses constitutes a feasible innovative option for limb-sparing surgery of locally advanced bone sarcomas and is associated with good functional results in short-term follow-up. Larger series with longer follow-up is necessary to evaluate the long-term implant survival.

## Data Availability

Detailed data is available from the authors by request.
